# Quantitative Targeted Absolute Proteomics for Better Characterization of an In Vitro Human Blood–Brain Barrier Model Derived from Hematopoietic Stem Cells

**DOI:** 10.3390/cells11243963

**Published:** 2022-12-08

**Authors:** Marie-Pierre Dehouck, Masanori Tachikawa, Yutaro Hoshi, Kotaro Omori, Claude-Alain Maurage, Guillaume Strecker, Lucie Dehouck, Marie-Christine Boucau, Yasuo Uchida, Fabien Gosselet, Tetsuya Terasaki, Yannis Karamanos

**Affiliations:** 1 Univ. Artois, UR 2465, Laboratoire de la Barrière Hémato-Encéphalique (LBHE), F-62300 Lens, France; 2 Graduate School of Biomedical Sciences, Tokushima University, 1-78-1 Shomachi, Tokushima 770-8505, Japan; 3 Division of Membrane Transport and Drug Targeting, Graduate School of Pharmaceutical Sciences, Tohoku University, Sendai 980-8577, Japan; 4 Pathology Department, CHRU Lille, 59037 Lille, France; 5 Intensive Care Unit, CHRU Lille, 59037 Lille, France; 6 School of Pharmacy, Faculty of Health Sciences, University of Eastern Finland, FI-70211 Kuopio, Finland

**Keywords:** blood–brain barrier, human in vitro models, human brain-like endothelial cells, brain microvessels, LC-MS/MS-based protein quantification, transporter

## Abstract

We previously developed an in vitro model of the human blood–brain barrier (BBB) based on the use of endothelial cells derived from CD34^+^-hematopoietic stem cells and cultured with brain pericytes. The purpose of the present study was to provide information on the protein expression levels of the transporters, receptors, tight junction/adherence junction molecules, and transporter-associated molecules of human brain-like endothelial cells (hBLECs). The absolute protein expression levels were determined by liquid chromatography–mass spectrometry-based quantitative targeted absolute proteomics and compared with those from human brain microvessels (hBMVs). The protein levels of CD144, CD147, MRP4, Annexin A6 and caveolin-1 showed more than 3-fold abundance in hBLECs, those of MCT1, Connexin 43, TfR1, and claudin-5 showed less than 3-fold differences, and the protein levels of other drug efflux transporters and nutrient transporters were less represented in hBLECs than in hBMVs. It is noteworthy that BCRP was more expressed than MDR1 in hBLECs, as this was the case for hBMVs. These results suggest that transports mediated by MCT1, TfR1, and claudin-5-related tight junction function reflect the in vivo BBB situation. The present study provided a better characterization of hBLECs and clarified the equivalence of the transport characteristics between in vitro BBB models and in vivo BBB models using LC-MS/MS-based protein quantification.

## 1. Introduction

Through a selective permeability process, the blood–brain barrier (BBB) plays a critical physiological role in maintaining brain homeostasis. However, it also constitutes a major hurdle for the drug delivery to the brain due to restricted and selective transport [1]. The BBB is localised in brain microvessel endothelial cells (EC) [2], and these cells, in the vicinity of (i) brain pericytes that share the same basement membrane as the endothelial cells (ECs), (ii) pedicellar extensions of astrocytes that continuously surround the cerebral microvessels, and (iii) neurons or neuronal progenitors in the perivascular brain parenchyma, form a dynamic barrier thanks to the occurrence of specialized proteins, namely, proteins of tight junctions, transporters, receptors, efflux pumps, etc. The BBB supported by ECs, with the surrounding cells previously cited, constitute the neurovascular unit (NVU) [3,4]. Numerous efforts have been made to develop in vitro BBB transport models to make it possible to predict in vivo BBB drug transport, as well as to study cellular transport mechanisms [5]. Since species differences between human and rodents have been demonstrated in terms of the protein expression levels of BBB transport-related proteins in isolated brain microvessels [6,7], the development of human-specific in vitro BBB models equivalent to in vivo human BBB models has proven to be important, and these models are being developed quickly [8]. Owing to an unpredictable supply, primary cultures from human brain endothelial cells are not an easily obtained source for the construction of in vitro BBB models. Thus, immortalized cell lines [9], inducible pluripotent stem cells [10,11], and hematopoietic stem cells [12,13] have gradually become the major sources for the establishment of in vitro human BBB models. Clarifying the similarities and differences in transport function between in vitro human BBB models and in vivo BBB is essential for the better prediction of BBB drug transport and understanding BBB transport systems in humans.

We developed a human BBB model by differentiating CD34^+^-cord blood hematopoietic stem cells into CD34^+^-endothelial cells and then co-cultivating them together with brain pericytes to induce BBB properties [14]. Tight junctional proteins such as Claudin-5/*CLDN5* and Occludin/*OCLN* were visualized at the cell–cell contact by immunofluorescence [15] and their functionality was highlighted by transmission electron microscopy (TEM), as they were able to block the passage of wheat germ agglutinin (WGA)–horseradish peroxidase. Paracellular transport was also assayed using small hydrophilic molecules, Lucifer yellow (LY) and sodium fluorescein (NaFlu). The permeability of the cells to LY and NaFlu was reduced 3- to 4-fold in the presence of the pericytes, while transendothelial electrical resistance (TEER) was increased 3-fold. These human brain-like endothelial cells (hBLECs) also express nutrient solute carrier (SLC) transporters for glucose (GLUT1/*SLC2A1*), amino acid L-arginine (CAT1/*SLC7A1*), monocarboxylic acids (MCT1/*SLC16A1*), and the receptor for transferrin (TfR1/*TFRC*), as well as ATP-binding cassette (ABC) transporters such as P-glycoprotein (MDR1/*ABCB1*) and BCRP/*ABCG2*, which play a key role in limiting drugs’ access to the brain from the blood. We have also clarified the functionality of these efflux transporters in several studies [14,16,17,18]. These results allowed a good qualitative and quantitative validation of hBLECs for a human in vitro BBB model [14,19,20].

The liquid chromatography–mass spectrometry (LC-MS/MS)-based quantitative targeted absolute proteomics (qTAP) experiment enabled us to clarify the species differences [7,21] and the equivalence between in vivo BBB and in vitro BBB models [22] in terms of transport function by comparing the absolute expression levels of the transport-related proteins in isolated brain microvessels (hBMVs) and immortalized human brain endothelial cells (hCMEC/D3) [23]. Thus, the purpose of the present study was to provide a better characterization of hBLECs by measuring the expression levels of BBB transport-related proteins, such as transporters, receptors, tight junction/adherence junction molecules and transporter-associated proteins, using a qTAP approach.

## 2. Materials and Methods

### 2.1. Human Samples

Brain occipital cortex samples from four Caucasian donors were obtained through a collaboration with hospital physicians and the multiple organ donation group of the University Hospital Centre (CHRU) of Lille. Donors hBMV1 (40 years old) and hBMV2 (43 years old) were males with no pathology and died accidentally. Donors hBMV3 (61 years old) and hBMV4 (83 years old) were females with diagnosed dementia. Due to formalities, the samples were taken between 12 h and 24 h post-mortem. Human cord blood was made available through an agreement between the Hospital of Béthune and Université d’Artois. Cord blood was used for the isolation of CD34^+^ stem cells. According to French legislation, all research protocols regarding the collection and use of human brain microvessels and cells derived from human cord blood were approved by the French ministry (CODECOH 2011-1321). In both cases, written informed consent was obtained from the families before entering the study. The protein quantification analyses with these human tissues and cells were also approved by the ethics committee of the Graduate School of Pharmaceutical Sciences, Tohoku University, Japan.

### 2.2. Isolation of Human Brain Microvessels (hBMVs)

The hBMVs were isolated using nylon mesh separation. All steps were performed using cold (4 °C) phosphate-buffered saline calcium that was magnesium-free (PBS-CMF: NaCl 8 g/L; KCl 0.2 g/L; KH_2_PO_4_ 0.2 g/L; Na_2_HPO_4_, 12H_2_O 2.87 g/L; pH 7.3–7.4). After removing the meninges, grey matter was cut into 5 mm^3^ fragments and washed with PBS-CMF. Cortex pieces suspended in 2 volumes of PBS-CMF were homogenized by 15 up-and-down motions in a glass homogenizer fitted with a glass pestle (0.152 mm clearance). Brain tissue homogenate was passed through a 180 µm nylon sieve, and most of the large vessels (venules and arterioles) were blocked. The filtrate containing microvessels enriched with capillaries was diluted and homogenized again with a second pestle (0.076 mm clearance) to remove brain tissue around the microvessels. The microvessels consisting mainly of brain capillaries were collected with a 60 µm nylon sieve and abundantly washed with PBS-CMF in order to remove cells debris. The microvessels were then collected directly on the 60 µm nylon sieve, suspended in a small volume of PBS-CMF, and stored in liquid nitrogen.

### 2.3. Cell Culture of Human Brain-like Endothelial Cells (hBLECs)

The BBB phenotype was induced in human umbilical cord blood stem cells through a BBB in vitro model consisting of a co-culture on either side of an insert, as described previously [14]. As the first step, CD34^+^-hematopoietic stem cells were isolated from cord blood and differentiated into endothelial cells, as previously described [24]. Then, these endothelial cells were seeded onto Matrigel (BD Biosciences, Franklin Lakes, NJ, USA, ref. 354230)-coated Transwell inserts (Corning, NY, USA, ref. CLS3419). As 100 × 10^6^ differentiated cells were necessary for one qTAP analysis, 75 mm Transwell inserts with 0.4 µm pore polycarbonate membranes were used. For one qTAP analysis, 10 inserts were seeded with 8 × 10^4^ endothelial cells/cm^2^ and co-cultured in the presence of 5 × 10^4^ brain pericytes/cm^2^ seeded at the bottom of the Petri dishes. The medium of the co-culture—EGM-2 composed of EGM basal medium (Lonza, Basel, Switzerland, ref. CC-3162) with all supplements except serum and gentamycin/amphotericin, supplemented with 2% (*v*/*v*) foetal calf serum, 50 µg/mL gentamycin (Biochrom AG, Berlin, Germany, ref. A-2712), and 1 ng/mL basic fibroblast growth factor—was renewed every 2 days. After 6 days of co-culture with pericytes, the endothelial cells differentiated into BBB endothelial cells (hBLECs).

hBLECs were collected by scraping them from the insert membranes after washing with cold (4 °C) phosphate-buffered saline with calcium and magnesium (PBS-Ca Mg: NaCl 8 g/L; KCl 0.2 g/L; KH_2_PO_4_ 0.2 g/L; Na_2_HPO_4_, 12H_2_O 2.87 g/L; CaCl_2_ 0.1 g/L; MgCl_2_, 6H_2_O 0.1 g/L; pH 7.3–7.4). All steps were realized on ice and without enzymatic digestion. The collected cells were centrifuged (230× *g*, 5 min, 4 °C) and the cell pellets were stored at −80 °C.

### 2.4. Preparation of Whole-Cell Lysate and Crude Membrane Fraction

The suspensions of hBMVs in PBS-CMF, or PBS-Ca Mg for hBLECs, as described above, were sonicated to prepare the whole-cell lysate. To prepare the crude membrane fraction from the hBLECs, the cell pellets were suspended in hypotonic buffer containing 10 mM Tris–HCl, 10 mM NaCl, and 1.5 mM MgCl_2_ at pH 7.4, and homogenized with a Teflon homogenizer followed by nitrogen cavitation at 600 psi for 15 min at 4 °C in a pressure vessel (Parr, Moline, IL, USA). The pellets were collected as the crude membrane fraction by the sequential centrifugations of the homogenate at 10,000× *g* for 10 min followed by 100,000× *g* for 40 min at 4 °C, and were suspended in the suspension buffer containing 10 mM Tris-HCl, pH 7.4, 250 mM sucrose. The protein concentration was determined by the Lowry method with bovine serum albumin as the standard (DC Protein Assay Kit, Bio-Rad, Hercules, CA, USA).

### 2.5. LC-MS/MS-Based qTAP

Protein expression amounts of the target molecules were simultaneously determined by means of multiplexed selective reaction monitoring (SRM) analysis, as described previously [25]. The LC-MS/MS-based qTAP analysis was performed using a mass spectrometer (TripleTOF5600, Sciex, Framingham, MA, USA) coupled with a nano-liquid chromatography system (Eksigent NanoLC Ultra 2D, Sciex, Framingham, MA, USA). The preparation of trypsin and lysyl endopeptidase-treated samples, the LC separation of peptides, and the detection/quantification of the target peptides were carried out as reported previously [26] with several modifications. The detailed method of the qTAP is described in the Supplementary Material. The sequences of the peptides to be quantified are summarized in Supplementary Table S1. The MS/MS transitions for the quantification of each peptide were set as reported previously [26,27]. A sample equivalent to 1 μg protein was injected onto the nano-liquid chromatography column (cHiPLC column ReproSil-Pur C18-AQ, 3 µm, 75 µm × 150 mm, Sciex). The mobile phases consisted of 0.1% formic acid in water (solution A) and 0.1% formic acid in acetonitrile (solution B). The peptides were separated and eluted from the column at a flow rate of 300 nL/min with a linear gradient as follows (mobile phase A:B): 100:0 for 40 min after injection for sample loading, 60:40 at 40 min, 10:90 at 41 min up to 50 min, 98:2 at 50.1 min and up to 80 min. The individual expression amount of each peptide was determined as an average ± S.E.M of three to five SRM transitions from one sample preparation in one analysis. The amount of GLUT3,14, CAT1, RFC1 and Connexin 43 in the crude membrane fraction of hBLEC was determined from one or two SRM transitions due to the low signals at the other transitions.

## 3. Results

The present study is the first investigation into the protein levels of 26 membrane proteins including transporters, receptors, tight junction/adherence junction molecules, and plasma membrane-associated molecules, including four marker proteins from hBLECs. Since hBLECs are used as in vitro BBB models, it was obvious to compare the obtained values with an in vivo BBB model. In addition to the possibility to compare the values with previously published data (hBMV_REF) [7], a new investigation was carried out with four donors, including two with no observed pathology (hBMV_NORM) and two with dementia (hBMV_PATHO). The complete list of the assayed proteins is presented in Table 1. In this investigation, a few more membrane proteins were quantified in addition to those reported in hBMV_REF, namely, Caveolin-1, Connexin 43, Annexin A6 and GFAP. Ezrin, Radixin and Moesin were exclusively assayed in hBLECs.

### 3.1. Analysis of the Protein Expression Levels in hBMVs and Comparison with the Previously Reported Quantification

**Figure 1:**
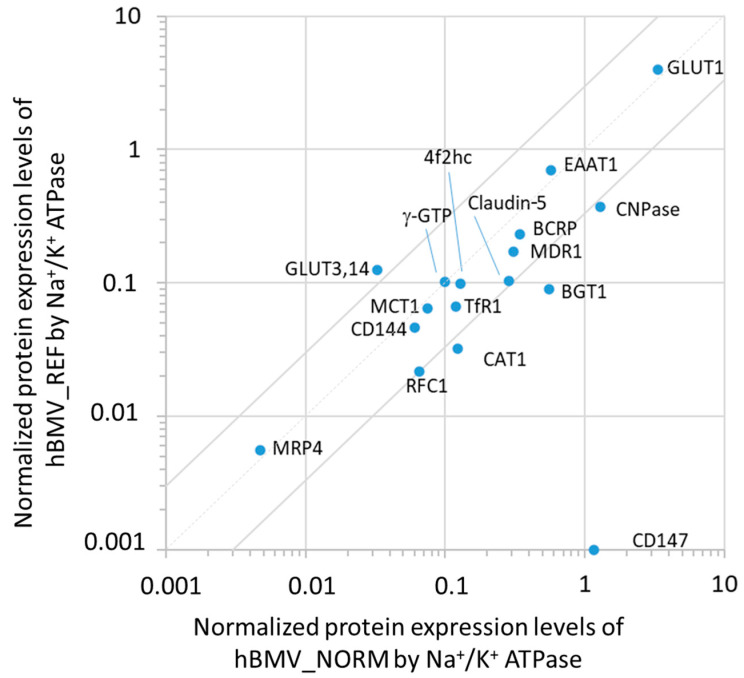
Comparison of the normalized protein expression levels in the isolated human brain microvessels obtained from the two independent donors with no pathology in this study (hBMV_NORM) with the previously reported protein expression levels in the isolated human brain microvessels from seven donors (hBMV_REF) [7]. The data sets of protein expression levels are from Table 1. The protein amounts were normalized by the level of Na^+^/K^+^ ATPase. The dot line passing through the origin represents the line of identity, and the grey lines represent 3-fold differences.

The average protein amounts in the whole-cell lysates of hBMV_NORM obtained from the two donors with no pathology were compared to the average protein amounts obtained from seven donors (hBMV_REF) reported previously [7]. The purity of the brain microvessel preparations was evaluated by measuring the protein expression amounts of marker proteins. The ratio between two of the hBMV marker proteins, γ-GTP and Na^+^/K^+^ ATPase [28], was equivalent to that previously reported [7]. This allowed us to normalize the protein amounts by the level of Na^+^/K^+^ ATPase (Figure 1).

The normalized levels of most of the proteins assayed showed less than 3-fold differences. In contrast, the levels of CNPase, CAT1 and BGT1 were 3.9-fold, 3.5-fold and 5.8-fold greater in hBMV_NORM, respectively. GLUT3,14 was 4-fold greater in hBMV_REF. CD147 was under the limit of quantification (<0.0828 fmol/µg protein) in hBMV_REF. The comparison of the values without normalization (Supplementary Figure S1), did not reveal other significant differences.

The comparison of hBMV_NORM with hBMV_PATHO (Supplementary Figure S2) did not reveal any significant differences except for MCT1 and CD147 (respectively 4-fold and 4.6-fold greater in hBMV_PATHO). It has been reported that both proteins are expressed at the BBB level and might be involved in degenerative processes [29,30].

### 3.2. Analysis of the Protein Expression Levels of hBLECs and Comparison with hBMV_NORM

**Figure 2:**
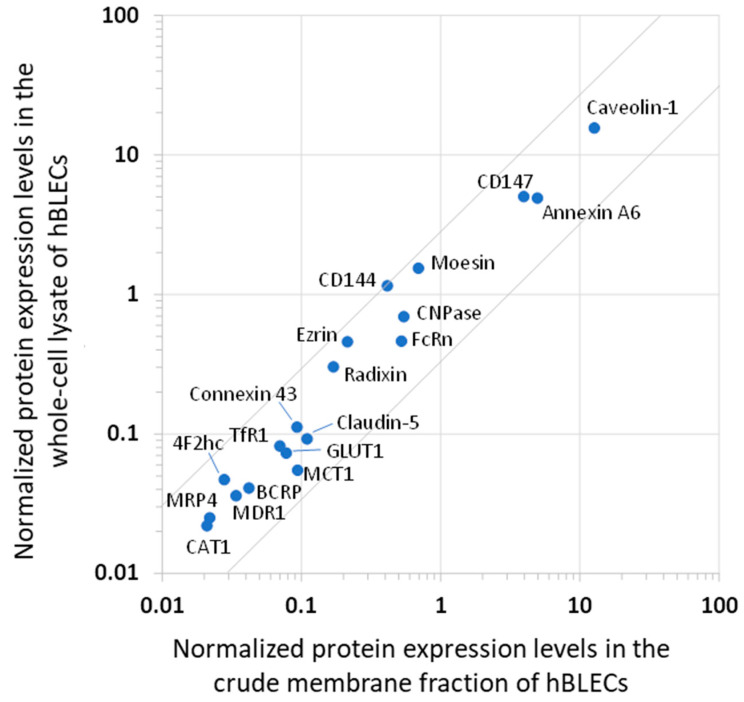
Comparison of the normalized protein expression levels of the crude membrane fraction and whole-cell lysate of human brain-like endothelial cells (hBLECs). The protein expression amounts were normalized by the level of Na^+^/K^+^ ATPase. The data on the protein expression levels are cited from Table 1. The grey lines represent 3-fold differences.

The expression levels of the membrane proteins from the hBLECs are listed in Table 1. With the exceptions of γ-GTP, EAAT1, GFAP, and BGT1 being under the limit of quantification, all the studied proteins were detected in the crude membrane fractions of the hBLECs at protein levels of 0.234 to 199 fmol/μg protein. With the exceptions of γ-GTP, EAAT1, GFAP BGT1, GLUT3,14, and RFC1, all the other studied proteins were detected in the whole-cell lysates of the hBLECs at the protein levels of 0.136 to 94.8 fmol/μg protein. The quantified values of membrane proteins, which were normalized by the level of Na^+^/K^+^ ATPases, were compared between the whole-cell lysates and the crude membrane fractions of the hBLECs (Figure 2). The result shows a good correlation of the normalized values of the whole-cell lysates and the crude membrane fractions within the 3-fold differences. Since the Na^+^/K^+^ ATPase proteins are predominantly localized in the plasma membrane, Na^+^/K^+^ ATPase can be used as a normalization marker for plasma membranes. Considering that the level of Na^+^/K^+^ ATPases in the crude membrane fractions was approximately 2.5-fold greater than that in the whole-cell lysates (Table 1), the plasma membranes would be concentrated by at least 2.5-fold in the crude membrane fractions compared to the whole-cell lysates.

**Figure 3:**
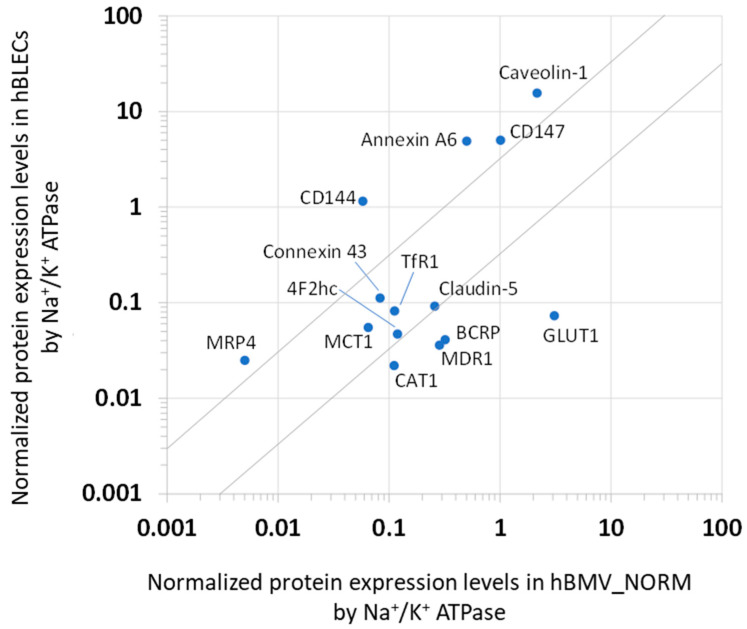
Comparison of the normalized protein expression levels in human brain-like endothelial cells (hBLECs) with those in the isolated human brain microvessels obtained from the two independent donors with no pathology in this study (hBMV_NORM). The protein expression amounts in the whole-cell lysates of hBLECs and hBMV_NORM were normalized by the level of Na^+^/K^+^ ATPase. The data on the protein expression levels are cited in Table 1. The grey lines represent 3-fold differences.

The comparison of the protein expression levels in the whole-cell lysate of the hBLECs with that of hBMV_NORM is presented in Figure 3. The normalized levels of Connexin 43, MCT1, TfR1, 4F2hc, and claudin-5 were within a 3-fold difference between hBLEC and hBMV_NORM. The proteins MDR1, BCRP, GLUT1, and CAT1 were less represented in the hBLECs. In contrast, the normalized levels of MRP4, CD144, CD147, Annexin A6 and caveolin-1 were more represented in the hBLECs (more than 3-fold differences).

## 4. Discussion

This is the first study investigating the protein expression of hBLECs by comparing the absolute protein expression levels of the membrane proteins involved in BBB transport with those in hBMV_NORM from two Caucasian donors with no observed pathology. We first compared the average protein levels in hBMV_NORM with the average protein levels in hBMV_REF obtained from seven donors, which we reported previously [7]. The comparison of hBMV_NORM with two Caucasian donors with dementia (hBMV_PATHO, Supplementary Figure S2) did not reveal any significant differences, except for CD147 and MCT1. The BBB-related proteins, e.g., MDR1, BCRP, EAAT1, GLUT1, GLUT3,14, RFC1, TfR1, claudin-5, CD144, and 4F2hc, and the marker of brain pericyte contamination ɣ-GTP [31,32,33], exhibited less than 3-fold differences. However, the protein level of BGT1, which is expressed in neurons and astrocytes [34], was 5.6-fold greater in hBMV_NORM than in hBMV_REF, and the level of CNPase, whose expression was strongly associated with myelin [35], was 3.7-fold greater in hBMV_NORM than in hBMV_REF. One possible explanation is that hBMV_NORM could be contaminated at greater levels by a population of brain parenchymal cells, although this would make little contribution to the expression levels of BBB-specific transporters. CD147 was under the limit of quantification in the previous study [7].

The protein expression levels in the hBLECs were compared to those in hBMV_NORM. It should first be mentioned that the comparison of the protein expression levels was made in between the hBLECs and the isolated microvessels. Considering that isolated microvessels contain not only endothelial cells, but also pericytes and glial cells which surround the endothelial cells, the best approach was to compare the hBLECs and the purified endothelial cells from the brain microvessels. However, due to the limitations of the human brain tissue samples obtained from each donor, it was technically difficult to obtain enough purified endothelial cells from human brain tissue for the present protein quantification analysis. This is a limitation of the present study. In the future, technology such as single-cell proteomics with greater sensitivity may solve the problems we faced in this study.

The present results indicate that the protein expression levels of membrane proteins, which are normalized by the level of Na^+^/K^+^ ATPases, show a good correlation at least partly in between the whole-cell lysates of hBMVs and hBLECs, as well as between the whole-cell lysates and crude membrane fractions of hBLECs. Several discussions may be needed about this point. The level of Na^+^/K^+^ ATPases per protein amount was greater in the whole-cell lysates of the hBMVs than the crude membrane fractions of the hBLECs (Table 1). We also found that the level of Na^+^/K^+^ ATPases per protein amount was 2- to 4-fold greater in the whole-cell lysates of the hBMVs (14.3–25.5 fmol/μg protein) than in the whole-cell lysates of the hBLECs (6.09 fmol/μg protein) (Table 1). Similar results are also seen in our previous report, showing that the level of Na^+^/K^+^ ATPases per protein amount in the whole-cell lysates was 5-fold greater in the hBMVs (35.1 fmol/μg protein [7]) than in in vitro brain microvessel endothelial cells (hCMECs/D3 cells) (7.31 fmol/μg protein [22]). One possibility to explain these findings is that the ratio of plasma membrane amount per protein amount of the whole-cell lysates might be different between in vivo hBMVs and in vitro human BBB cell lines such as hBLECs and hCMECs/D3 cells. Our previous findings have also shown that the levels of membrane proteins, which are normalized by the level of Na^+^/K^+^ ATPase, exhibit a good correlation between the whole-cell lysates of hBMVs and the plasma membrane fractions of hCMECs/D3 cells [22]. This suggests that the level of Na^+^/K^+^ ATPase can be used to normalize not only the extraction efficiency of plasma membranes, but also the difference in the ratio of plasma membrane amount in the whole-cell lysates of in vivo hBMVs and in vitro hBLECs.

The protein levels of EAAT1, GFAP and BGT1, which are expressed in brain parenchymal cells such as neurons and glial cells [34,36], are under the detection limit in hBLECs, whereas those proteins were detected in hBMV_NORM. In addition, γ-GTP which is a pericyte marker, is present in hBMV_NORM, but not in hBLECs. Altogether, these results would support the notion that brain parenchymal cells and brain pericytes are, to an extent, contained in hBMV_NORM. In our study, CNPase, which is usually used as a marker of oligodendrocytes, was detected in hBMV_NORM, and hBLEC. This is not surprising since CNPase has previously been detected in brain endothelial cells [37], and this is also the case for CD147, which has been reported to be exclusively expressed in endothelial cells [30].

The results obtained for hBLECs indicate that the protein expressions of drug efflux transporters such as MDR1 and BCRP, the glucose transporter GLUT1, and CAT1 were lower compared to the in vivo situation. The BBB efflux transporters MDR1 and BCRP constitute some of the major hurdles in drug delivery to the brain [38]. One possible explanation for MDR1 and BCRP is that astrocytes and brain pericytes, present in microvessels, also express these transporters, as previously demonstrated [39,40]. This conclusion is strengthened by the fact that the functionality of these transporters has been largely demonstrated in our recent studies using this BBB model [14,16,18,41]. Interestingly, with the present study, we now know that BCRP is more expressed than MDR1 in hBLECs, as previously reported in hBMV_REF [7].

We also report that the expression of GLUT1, which facilitates the transport of glucose to the brain parenchyma [42], decreases sharply in hBLECs. Since its expression is regulated by glucose concentration [43], its protein expression would have been affected by the cell culture conditions [18] and the presence of brain pericytes in the present study.

On the other hand, the protein levels of monocarboxylate transporter MCT1, transcytotic transferrin receptor TfR1, and tight junction protein claudin-5 are almost comparable between hBLECs and hBMVs, in the range of 3-fold differences. These results suggest that hBLECs could mostly reflect the in vivo BBB transport characteristics of monocarboxylate, transferrin, and claudin-5-related tight junction function [44]. This conclusion is in line with our previous studies using this human BBB model, demonstrating that nanoparticles designed to interact with TfR1 are able to be transcytosed more efficiently through hBLECs than the control nanoparticles [45,46].

Furthermore, the expressions of the adherence molecule CD144 [47], as well as the transporter-associated proteins CD147, caveolin-1, and annexin A6, are upregulated in hBLECs compared to microvessels. The present results also indicate the expressions in the crude membrane fractions of hBLECs, as well as proteins such as ezrin, radixin, and moesin, which are required for plasma membrane localization by linking actin filaments to plasma membrane proteins [48]. The protein expression levels of ezrin, radixin, and moesin are comparable to those of CD144 in the crude membrane fractions of hBLECs. A differential proteomics approach demonstrated that these three proteins were increased in brain capillary endothelial cells with re-induced BBB functions [49]. It has also been reported that CD147 is essential for MCT1 translocation to the cell surface and its transport activity [50]. Caveolin-1 and annexin A6 contribute to the organization of membrane microdomains, e.g., cholesterol-rich lipid rafts [51]. Our recent report has demonstrated that the phosphorylation of caveolin-1 induces the internalization of the MDR1 protein in human brain microvascular endothelial cells (hCMECs/D3 cells), which leads to a decrease in MDR1-mediated cortisol efflux transport [52]. We have also found that ezrin, radixin, and moesin play distinct roles in the plasma membrane localization of transporters such as MDR1, BCRP, and GLUT1, and transporter-related functions [53]. Taken together, these findings suggest that the CD144-mediated adherence function, and the CD144-, caveolin-1- and annexin A6-mediated organization of plasma membrane proteins, including transporters, may be overestimated in hBLECs compared to the in vivo human BBB model.

In the present study, the presence of neonatal Fc receptor (FcRn) in hBLECs was also demonstrated. FcRn has an uphill-type expression in rat developing brain capillaries [54]. The expression of FcRn has been initially described in rat brain microvasculature and choroid plexus epithelium using confocal microscopy and an antibody to the rat neonatal FcRn [55], and colocalised with GLUT1, thus highlighting its expression in the capillary endothelium. FcRn is responsible for the extended persistence of IgG and other Fc-conjugated proteins in the serum. Many therapeutic approaches that involve FcRn have been suggested to strengthen or weaken the FcRn-IgG interaction [56]. Thus, hBLECs seem suitable for studying the involvement of FcRn in reverse transcytosis in the brain to blood direction, as suggested [55].

In conclusion, the present study provided a better characterization of transport systems in hBLECs by comparing the levels of transporter-related proteins in hBLECs and isolated vessels. This provides references for in vitro BBB models for the development of brain-targeting therapeutics, in particular when targeting TfR1.

## Figures and Tables

**Table 1:** Protein expression levels of transporters, receptors, tight junctions/adhesion molecules, plasma membrane-associated molecules, and marker proteins in hematopoietic stem cell-derived human brain-like endothelial cells (hBLECs) in isolated human brain microvessels with no pathology (hBMV_NORM) or with dementia (hBMV_PATHO), with previously published values taken as the reference values [7] (hBMV_REF).

Protein Expression Levels										
Protein Name/*Gene Name*	hBLEC Crude Membrane Fraction		hBLEC Whole-Cell Lysate		hBMV_NORM Whole-Cell Lysate		hBMV_PATHO Whole-Cell Lysate		hBMV_REF Whole-Cell Lysate	
	Value ^a^ (fmol/μg Protein)	Normalized ^b^	Quadratic Mean ^c^ (fmol/μg Protein)	Normalized ^b^	Quadratic Mean ^d^ (fmol/μg Protein)	Normalized ^b^	Quadratic Mean ^d^ (fmol/μg Protein)	Normalized ^b^	Value ^e^ (fmol/μg Protein)	Normalized ^b^
*Transporters or Receptors*										
MDR1/*ABCB1*	0.536 ± 0.024	0.034	0.221 ± 0.065	0.036	7.25 ± 2.57	0.285	5.44 ± 2.23	0.380	6.06 ± 1.69	0.173
MRP4/*ABCC4*	0.324 ± 0.015	0.022	0.152 ± 0.037	0.025	0.118 ± 0.001	0.005	0.072 ± 0.087	0.005	0.195 ± 0.069	0.006
BCRP/*ABCG2*	0.659 ± 0.057	0.042	0.251 ± 0.014	0.041	8.15 ± 2.45	0.320	5.44 ± 2.41	0.379	8.14 ± 2.26	0.232
EAAT1/*SLC1A3*	U.L.Q. (<0.173)	-	U.L.Q. (<0.114)	-	14.2 ± 1.1	0.559	10.4 ± 2.2	0.728	24.5 ± 12.5	0.698
GLUT1/*SLC2A1*	1.22 ± 0.11	0.078	0.443 ± 0.068	0.073	78.6 ± 23.5	3.09	55.2 ± 21.2	3.85	139 ± 46	3.960
BGT1/*SLC6A12*	U.L.Q. (<1.320)	-	U.L.Q. (< 0.989)	-	13.3 ± 2.3	0.523	6.73 ± 1.42	0.470	3.16 ± 0.94	0.090
GLUT3,14/*SLC2A3,14*	0.234 *	0.015	N.D.	-	0.800 ± 0.048	0.031	1.06 ± 0.49	0.074	4.40 ± 1.00	0.125
CAT1/*SLC7A1*	0.329 *	0.021	0.136 ± 0.041	0.022	2.84 ± 1.73	0.111	3.44 ± 2.23	0.240	1.13 ± 0.18	0.032
MCT1/*SLC16A1*	1.46 ± 0.15	0.094	0.335 ± 0.043	0.055	1.65 ± 1.61	0.065	3.77 ± 1.66	0.263	2.27 ± 0.85	0.065
RFC1/*SLC19A1*	0.248 **	0.016	N.D.	-	1.61 ± 0.12	0.063	0.515 ± 0.25	0.036	0.763 ± 0.041	0.022
TfR1/*TFRC*	1.17 ± 0.09	0.075	0.500 ± 0.146	0.082	2.86 ± 0.85	0.112	2.05 ± 0.35	0.143	2.34 ± 0.76	0.067
FcRn/*FCGRT*	8.16 ± 0.47	0.523	2.82 ± 0.35	0.463	N.D.	-	N.D.	-	N.D.	-
*Others*										
Claudin-5/*CLDN5*	1.72 ± 0.14	0.110	0.562 ± 0.043	0.092	6.59 ± 3.49	0.258	4.05 ± 1.09	0.283	3.62 ± 0.96	0.103
CD144/*CDH5*	6.47 ± 0.20	0.415	7.02 ± 1.00	1.15	1.48 ± 0.17	0.058	1.25 ± 0.58	0.087	1.61 ± 0.43	0.046
4F2hc/*SLC3A2*	0.438 ± 0.020	0.028	0.288 ± 0.040	0.047	3.0 ± 0.89	0.119	2.01 ± 1.24	0.140	3.47 ± 0.83	0.099
CD147/*BSG*	61.8 ± 2.2	3.96	30.6 ± 2.5	5.02	25.6 ± 22.8	1.01	66.4 ± 11.5	4.64	U.L.Q. (<0.083)	-
Caveolin-1/*CAV1*	199 ± 2	12.7	94.8 ± 8.7	15.6	55.0 ± 20.4	2.16	40.1 ± 7.8	2.79	N.D.	-
Connexin 43/*CX43*	1.46 **	0.093	0.682 ±0.099	0.112	2.12 ± 0.67	0.083	1.74 ± 0.15	0.121	N.D.	-
Annexin A6/*ANXA6*	77.4 ± 1.9	4.96	29.8 ± 4.57	4.90	12.8 ± 5.4	0.502	6.34 ± 0.99	0.442	N.D.	-
Ezrin/*EZR*	3.34 ± 0.21	0.214	2.79 ± 0.65	0.458	N.D.	-	N.D.	-	N.D.	-
Radixin/*RDX*	2.65 ± 0.73	0.170	1.84 ± 0.53	0.302	N.D.	-	N.D.	-	N.D.	-
Moesin/*MSN*	10.8 ± 0.3	0.692	9.36 ± 1.87	1.54	N.D.	-	N.D.	-	N.D.	-
*Markers proteins*										
γ-GTP/*GGT*	U.L.Q. (<0.127)	-	U.L.Q. (<0.098)	-	2.37 ± 0.79	0.093	1.80 ± 0.19	0.1126	3.57 ± 0.71	0.102-
GFAP/*GFAP*	U.L.Q. (<0.095)	-	U.L.Q. (<0.080)	-	5.38 ± 2.72	0.211	11.2 ± 0.2	0.781	N.D.	-
CNPase/*CNP*	8.49 ± 0.46	0.544	4.22 ± 0.59	0.693	36.9 ± 41.1	1.45	18.1 ± 1.9	1.26	12.96 ± 4.12	0.369
Na^+^/K^+^ ATPase	15.6 ± 1.2	1	6.09 ± 0.83	1	25.5 ± 6.4	1	14.3 ± 14.8	1	35.1 ± 12.6	1

U.L.Q. represents under the limit of quantification, and the detection limit of quantification is indicated as the value of detection limit. N.D. represents no data available. ^a^ Each quantitative value, except for the values marked by asterisks, represents the mean ± S.E.M. of the quantitative values determined from three to five MS/MS transitions in one analysis. A single asterisk represents the mean of quantitative values obtained from two transitions. Double asterisks represent the quantitative value obtained from one transition. The reliability of the protein expression values calculated from one or two transition(s) is lower than that of values obtained from three to five transitions. ^b^ Values normalized by the Na^+^/K^+^ ATPase value without considering S.D. or S.E.M. ^c^ Quadratic mean ± S.D. of the values obtained from the three independent samples of hBLEC. ^d^ Quadratic mean ± S.D. of the values obtained from the two donors with no pathology, hBMV1 and hBMV2, or from the two donors with dementia, hBMV3 and hBMV4 (individual values are presented in the Supplementary Table S2). ^e^ The data for the average of seven donors were taken from [7] and represent mean ± S.D.

## Data Availability

The datasets used and/or analysed during the current study are available from the corresponding author on reasonable request.

## Supplementary Materials

The following supporting information can be downloaded at: https://www.mdpi.com/article/10.3390/cells11243963/s1, Figure S1: Comparison of the average protein expression levels in isolated human brain microvessels from two donors in this study (hBMV_NORM) and the previously reported values as the average protein expression levels in the isolated human brain microvessels from seven donors (hBMV_REF); Figure S2: Comparison of the average protein expression levels in the isolated human brain microvessels from the two donors with no observed pathology (hBMV_NORM), and those from the two donors with dementia (hBMV_PATHO); Table S1: Peptide probes to quantify the target molecules; Table S2: Protein expression levels of transporters, receptors, tight junctions/adhesion molecules, plasma membrane-associated molecules, and markers in isolated human brain microvessels from the brain cortexes of four independent donors (hBMV1 to hBMV4); Supplementary Methods: The detailed method of quantitative targeted absolute proteomics (qTAP).
