# Supplementary material for: Quantitative Targeted Absolute Proteomics for Better Characterization of an In Vitro Human Blood–Brain Barrier Model Derived from Hematopoietic Stem Cells
Source: Cells. 2022 Dec 8;11(24):3963. doi: 10.3390/cells11243963 (PMC9776576; doi:10.3390/cells11243963)
Supplement: Supplementary file 1 [file cells-11-03963-s001.zip › cells-2001452-SM.pdf]

cells

MDPI

Article

Quantitative Targeted Absolute Proteomics for Better Characterization of an In Vitro Human Blood–Brain barrier Model Derived from Hematopoietic Stem Cells

Marie-Pierre Dehouck [1,*,†], Masanori Tachikawa [2,3,†], Yutaro Hoshi [3], Kotaro Omori [3], Claude-Alain Maurage [4], Guillaume Strecker [5], Lucie Dehouck [1], Marie-Christine Boucau [1], Yasuo Uchida [3], Fabien Gosselet [1], Tetsuya Terasaki [3,6,†] and Yannis Karamanos [1,*,†]

[1] Univ. Artois, UR 2465, Laboratoire de la Barrière Hémato-Encéphalique (LBHE), F-62300 Lens, France
[2] Graduate School of Biomedical Sciences, Tokushima University, 1-78-1 Shomachi, Tokushima 770-8505, Japan
[3] Division of Membrane Transport and Drug Targeting, Graduate School of Pharmaceutical Sciences, Tohoku University, Sendai 980-8577, Japan
[4] Pathology Department, CHRU Lille, 59037 Lille, France
[5] Intensive Care Unit, CHRU Lille, 59037 Lille, France
[6] School of Pharmacy, Faculty of Health Sciences, University of Eastern Finland, FI-70211 Kuopio, Finland
* Correspondence: mpierre.dehouck@univ-artois.fr (M.-P.D.); yannis.karamanos@univ-artois.fr (Y.K.)
† These authors contributed equally to this work.

Citation: Dehouck, M.-P.; Tachikawa, M.; Hoshi, Y.; Omori, K.; Maurage, C.-A.; Strecker, G.; Dehouck, L.; Boucau, M.-C.; Uchida, Y.; Gosselet, F.; et al. Quantitative Targeted Absolute Proteomics for Better Characterization of an In Vitro Human Blood–Brain Barrier Model Derived from Hematopoietic Stem Cells. *Cells* **2022**, *11*, 3963. https://doi.org/10.3390/cells11243963

Academic Editor(s): Julia Schumann and Michael Cross

Received: 15 October 2022
Accepted: 5 December 2022
Published: 8 December 2022

Publisher's Note: MDPI stays neutral with regard to jurisdictional claims in published maps and institutional affiliations.

Copyright: © 2022 by the authors. Submitted for possible open access publication under the terms and conditions of the Creative Commons Attribution (CC BY) license (https://creativecommons.org/licenses/by/4.0/).

Supplementary Materials

Figure S1: Comparison of the average protein expression levels in the isolated human brain microvessels from two donors in this study (hBMV_NORM), and the previously reported values as the average protein expression levels in the isolated human brain microvessels from seven donors (hBMV_REF);

Figure S2: Comparison of the average protein expression levels in the isolated human brain microvessels from the two donors with no observed pathology (hBMV_NORM), and those from the two donors with dementia (hBMV_PATHO).;

Table S1: Peptide probes to quantify the target molecules;

Table S2: Protein expression levels of transporters, receptors, tight junctions/adhesion molecules, plasma membrane-associated molecules, and markers in isolated human brain microvessels from the brain cortexes of four independent donors (hBMV1 to hBMV4);

Supplementary Methods: The detailed method of quantitative Targeted Absolute Proteomics (qTAP)

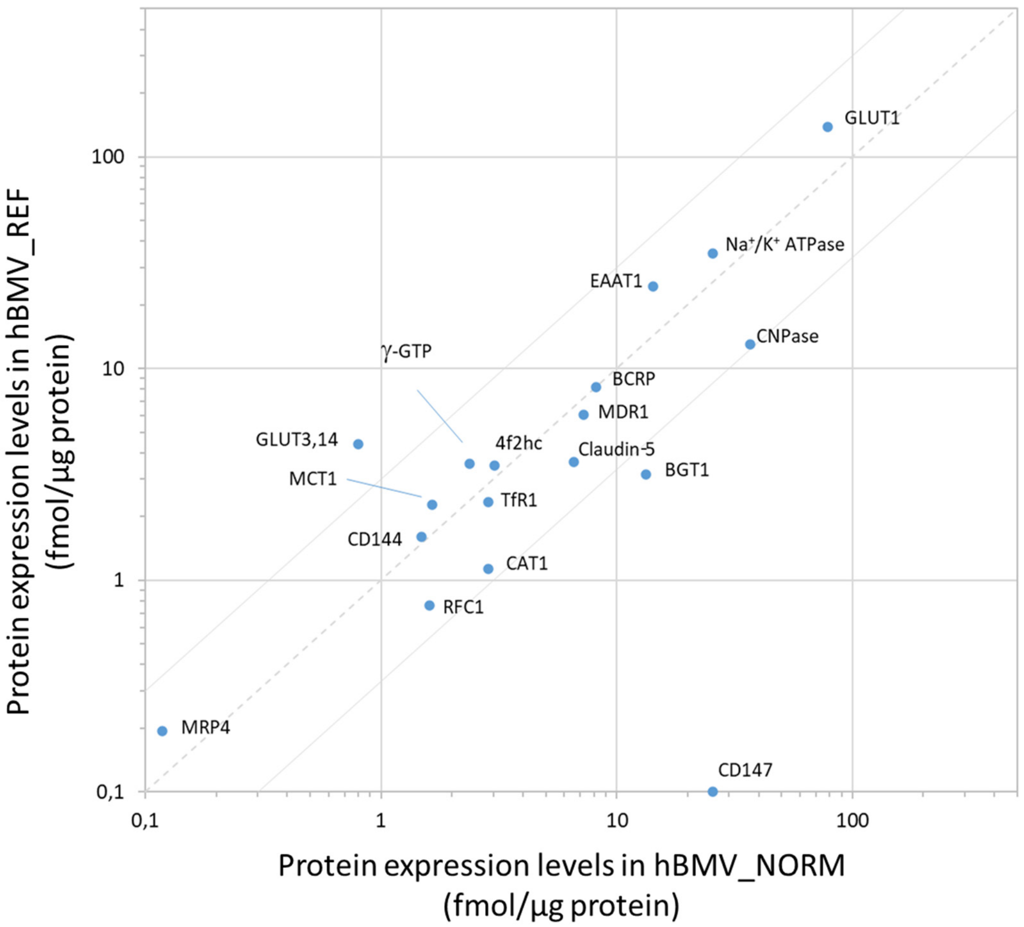

Supplementary Figure S1 Comparison of the average protein expression levels in the isolated human brain microvessels from two donors in this study (hBMV_NORM), and the previously reported values as the average protein expression levels in the isolated human brain microvessels from seven donors (hBMV_REF) [4]. The data set of protein levels obtained in the present study is the average of the values cited in Table 1. The dot line passing through the origin represents the line of identity, and the grey lines represent 3-fold differences.

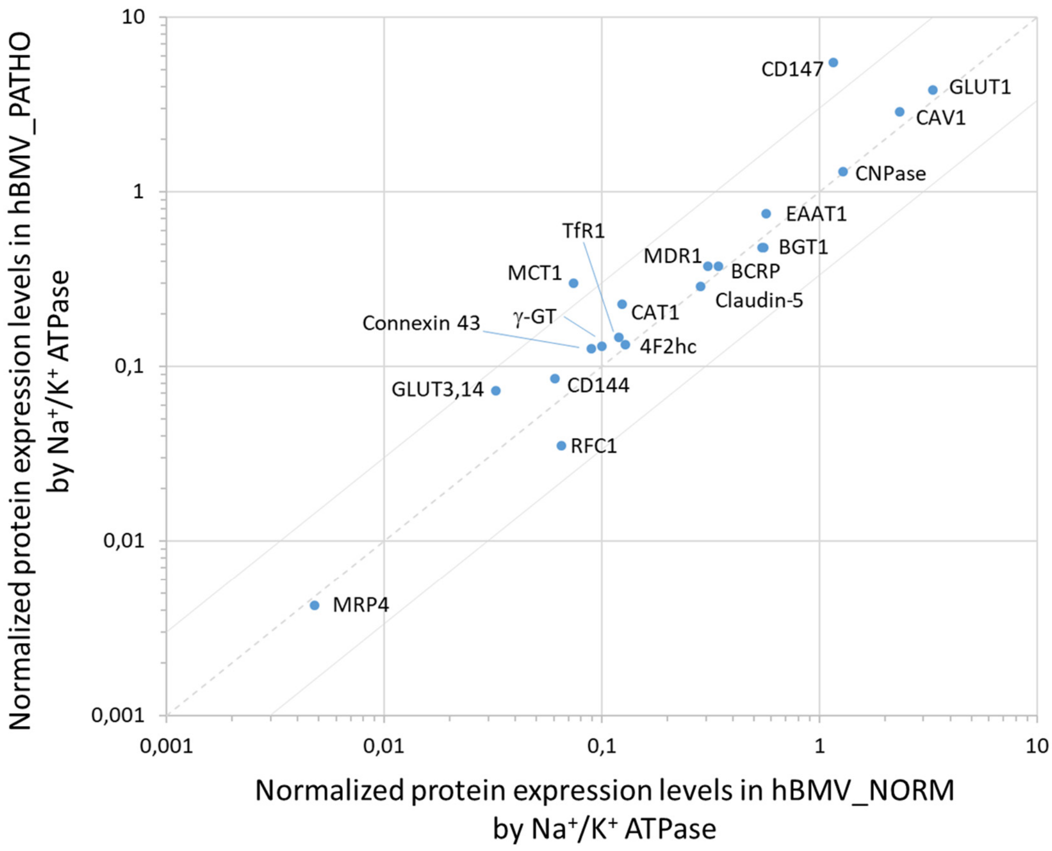

Supplementary Figure S2 Comparison of the average protein expression levels in the isolated human brain microvessels from the two donors with no observed pathology (hBMV_NORM), and those from the two donors with dementia (hBMV_PATHO). The data sets are from the supplementary Table S1. The dot line passing through the origin represents the line of identity, and the grey lines represent 3-fold differences.

Supplementary Table S1 Peptide probes to quantify the target molecules

Gene Symbol	Alias	St or Is	Probe Sequence	Number of AA	Position of stable isotope-labeled AA from N-terminal	Stable isotope-labeled AA
ABCB1	MDR1	St	FYDPLAGK	8		
		Is	FYDPLAGK	8	6	A
ABCC4	MRP4	St	APVLFFDR	8		
		Is	APVLFFDR	8	4	L
ABCG2	BCRP	St	SSLLDVLAAR	10		
		Is	SSLLDVLAAR	10	7	L
SLC1A3	EAAT1	St	VTAADAFLDLIR	12		
		Is	VTAADAFLDLIR	12	11	I
SLC2A1	GLUT1	St	TFDEIASGFR	10		
		Is	TFDEIASGFR	10	6	A
SLC2A3,14	GLUT3,14	St	QVTVLELFR	9		
		Is	QVTVLELFR	9	8	F
SLC3A2	4F2hc	St	ADLLLSTQPGR	11		
		Is	ADLLLSTQPGR	11	9	P
SLC6A12	BGT1	St	NFGPSPTR	8		
		Is	NFGPSPTR	8	6	P
SLC7A1	CAT1	St	FLANVNDR	8		
		Is	FLANVNDR	8	5	V
SLC16A1	MCT1	St	SITVFFK	7		
		Is	SITVFFK	7	6	F
SLC19A1	RFC1	St	QPPAQGLR	8		
		Is	QPPAQGLR	8	7	L
	TfR1	St	SGVGTALLLK	10		
		Is	SGVGTALLLK	10	9	L
	FcRn	St	GNLEWK	6		
		Is	GNLEWK	6	6	K
	Claudin-5	St	EFYDPSVPVSQK	12		
		Is	EFYDPSVPVSQK	12	9	V
	CD144	St	YTFVVPEDTR	10		
		Is	YTFVVPEDTR	10	6	P
	CD147	St	EDALPGQK	8		
		Is	EDALPGQK	8	5	P
	Caveolin-1	St	HLNDDVVK	8		
		Is	HLNDDVVK	8	8	K
	Connexin 43	St	SDPYHATSGALSPAK	15		

		Is	SDPYHATSGALSPAK	15	15	K
	Annexin A6	St	ALIEILATR	9		
		Is	ALIEILATR	9	9	R
	Ezrin	St	FYPEDVAEELIQDITQK	17		
		Is	FYPEDVAEELIQDITQK	17	17	K
	Radixin	St	GYSTWLK	7		
		Is	GYSTWLK	7	7	K
	Moesin	St	GFSTWLK	7		
		Is	GFSTWLK	7	7	K
	gamma-GTP	St	LFQPSIQLAR	10		
		Is	LFQPSIQLAR	10	8	L
	GFAP	St	FASYIEK	7		
		Is	FASYIEK	7	5	I
	CNPase	St	LSPTDNLPR	9		
		Is	LSPTDNLPR	9	8	P
Na^+/K^+ ATPase (α1, α2, α3 sub-units)	Na^+/K^+ ATPase (α1, α2, α3 subu-nits)	St	AAVPDAVGK	9		
		Is	AAVPDAVGK	9	7	V

^{13}C and ^{15}N were used for amino acid labeling in the internal standard peptides (stable isotope-labeled peptides). The amino acid sequence of probe peptide is specific for the target molecule. AA, amino acid; St, standard; Is, internal standard.

Supplementary Table S2. Protein expression levels of transporters, receptors, tight junctions/adhesion molecules, plasma membrane-associated molecules, and markers in isolated human brain microvessels from the brain cortexes of two independent donors with no pathology (hBMV1 and hBMV2), and two independent donors with dementia (hBMV3 and hBMV4)

Protein name/ *Gene name*	**Protein expression levels**							
	**hBMV1** Whole cell lysate		**hBMV2** Whole cell lysate		**hBMV3** Whole cell lysate		**hBMV4** Whole cell lysate	
	Value[a] (fmol/µg protein)	Normalized[b]	Value[a] (fmol/µg protein)	Normalized[b]	Value[a] (fmol/µg protein)	Normalized[b]	Value[a] (fmol/µg protein)	Normalized[b]
Transporters or Receptors								
MDR1/*ABCB1*	5.42 ± 0.41	0.181	9.06 ± 0.40	0.431	3.86 ± 0.10	0.271	7.02 ± 0.19	0.336
MRP4/*ABCC4*	0.117*	0.004	0.118*	0.006	0.011**	0.001	0.133 ± 0.011	0.008
BCRP/*ABCG2*	6.42 ± 0.34	0.214	9.88 ± 0.23	0.470	3.74 ± 0.07	0.341	7.14 ± 0.16	0.403
EAAT1/*SLC1A3*	15.0 ± 0.6	0.500	13.4 ± 0.3	0.638	8.88 ± 0.35	0.811	12.0 ± 0.2	0.677
GLUT1/*SLC2A1*	61.9 ± 2.1	2.063	95.2 ± 2.3	4.533	40.2 ± 0.9	3.673	70.2 ± 0.5	3.965
BGT1/*SLC6A12*	11.7 ± 0.4	0.390	15.0 ± 0.4	0.714	5.72 ± 0.26	0.523	7.73 ± 0.36	0.436
GLUT3,14/ *SLC2A3,14*	0.765 ± 0.043	0.025	0.833 ± 0.049	0.040	0.713 ± 0.062	0.065	1.41 ± 0.07	0.079
CAT1/*SLC7A1*	1.60 ± 0.25	0.053	4.04 ± 0.42	0.192	1.85 ± 0.23	0.169	5.02 ± 0.24	0.283
MCT1/*SLC16A1*	0.507*	0.017	2.76 ± 0.37	0.131	4.93 ± 0.22	0.451	2.59 ± 0.20	0.146
RFC1/*SLC19A1*	1.51 ± 0.22	0.050	1.68 ± 0.20	0.080	0.334**	0.031	0.696**	0.039*
TfR1/*TFRC*	2.22 ± 0.39	0.074	3.46 ± 0.08	0.165	1.80 ± 0.05	0.165	2.29 ± 0.16	0.129
FcRn/*FCGRT*	N.D.	-	N.D.	-	N.D.	-	N.D.	-
Others								
Claudin-5/*CLDN5*	4.08 ± 0.51	0.136	9.03 ± 0.66	0.430	3.28 ± 0.13	0.300	4.82 ± 0.08	0.272
CD144/*CDH5*	1.34 ± 0.25	0.045	1.60 ± 0.08	0.076	0.837 ± 0.080	0.076	1.66 ± 0.06	0.094

4F2hc/*SLC3A2*	2.41 ± 0.10	0.080	3.67 ± 0.06	0.175	1.13 ± 0.09	0.103	2.89 ± 0.04	0.163
CD147/*BSG*	9.45 ± 0.92	0.315	41.8 ± 0.7	1.990	97.9 ± 0.47	8.943	34.9 ± 1.5	1.971
Caveolin-1/*CAV1*	40.6 ± 0.9	1.354	69.4 ± 0.4	3.311	34.6 ± 0.2	3.160	45.6 ± 1.1	2.574
Connexin 43/*CX43*	1.64**	0.055	2.58 ± 0.25	0.123	1.63**	0.149	1.84 ± 0.07	0.104
Annexin A6/*ANXA6*	8.98 ± 0.14	0.299	16.6 ± 0.7	0.790	7.02 ± 0.61	0.641	5.62 ± 0.37	0.317
Ezrin/*EZR*	N.D.	-	N.D.	-	N.D.	-	N.D.	-
Radixin/*RDX*	N.D.	-	N.D.	-	N.D.	-	N.D.	-
Moesin/*MSN*	N.D.	-	N.D.	-	N.D.	-	N.D.	-
Markers proteins								
γ-GTP/*GGT*	1.81 ± 0.08	0.060	2.92 ± 0.20	0.139	1.67 ± 0.08	.152	1.93± 0.18	.109
GFAP/*GFAP*	7.27 ± 0.69	0.242	3.33 ± 0.93	0.158	11.1 ± 0.5	1.02	11.3 ± 0.9	0.638
CNPase/*CNP*	65.9 ± 1.3	2.20	7.81 ± 0.06	0.371	15.4 ± 0.2	1.41	20.9 ± 0.7	1.18
Na^+/K^+ ATPase	30.0 ± 0.9	1	21.0 ± 0.2	1	10.9 ± 0.2	1	17.7 ± 0.3	1

[a] Each quantitative value, except for the values marked by asterisks, represents the mean ± S.E.M. of the quantitative values determined from three to five MS/MS transitions in one analysis. A single asterisk represents the mean of quantitative values obtained from two transitions. Double asterisks represent the quantitative value obtained from one transition. The reliability of the protein expression values calculated from one or two transition(s) is lower than that of values obtained from three to five transitions. U.L.Q. represents under the limit of quantification and the detection limit of quantification was indicated as the value of detection limit. N.D. represents no data available.

[b] values normalized by the quadratic mean of Na^+/K^+ ATPase

Supplementary Methods: The detailed method of quantitative Targeted Absolute Proteomics (qTAP)

1. Lysyl endopeptidase and trypsin digestion.
The whole cell lysate and crude membrane fraction were suspended in denaturing buffer (7 M guanidine hydrochloride, 500 mM Tris–HCl (pH 8.5) and 10 mM EDTA) to solubilize and denature proteins, and then treated with dithiothreitol at room temperature for 60 min followed by iodoacetamide at room temperature for 60 min in darkness to reduce and alkylate the proteins. The proteins were then purified by a methanol-chloroform precipitation method. The resultant precipitates were dissolved in urea buffer (6 M urea and 100 mM Tris–HCl (pH 8.5)), diluted fivefold with 100 mM Tris–HCl (pH 8.5), and sonicated in an ultrasonic bath containing ice to ensure complete dissolution. ProteaseMax surfactant (Promega, Madison, Wisconsin) and lysyl endopeptidase (LysC; Wako Pure Chemical Industries, Osaka, Japan) were added at 0.05% final concentration and an enzyme/substrate ratio of 1:100, respectively, and the mixture was incubated at room temperature for 3 h, followed by digestion with TPCK-treated trypsin (Promega) at an enzyme/substrate ratio of 1:100 at 37°C for 16 h. The resulting peptide samples were acidified with trifluoroacetic acid.

2. Clean-up of peptide samples for LC-MS/MS analysis.
For the calibration curve, tryptic digests were obtained from 10 μg protein of E. coli lysate and spiked with standard peptides (0, 1, 2.5, 5, 10, 25, 50, 100, 500, 1000, or 2000 fmol for the points of 0, 0.1, 0.25, 0.5, 1, 2.5, 5, 10, 50, 100, or 200 fmol/μg protein, respectively) and 500 fmol of internal standard peptides. The internal standard peptide had an identical amino acid sequence to that of the corresponding standard peptide, and one amino acid residue of each internal standard was labeled with stable isotopes (13C and 15N) (Supplementary Table S1). For the whole cell lysate and crude membrane fraction, the tryptic digests obtained from 10 μg protein of plasma membrane fractions were spiked with 500 fmol of internal standard peptides. All samples were cleaned up by using GL-tip GC and SDB (GL Science Inc., Tokyo, Japan), according to the manufacturer's protocol. The resultant eluates were evaporated by centrifugation under vacuum for 1 h with a CC-105 centrifugal concentrator (low heat mode; TOMY, Tokyo, Japan), and the residues were reconstituted in 0.1% formic acid/water. One-tenth volume was injected into the LC-MS/MS system: on the assumption that there was no loss of peptides during the clean-up, each injection for the calibration curve included 0, 0.1, 0.25, 0.5, 1, 2.5, 5, 10, 50, 100, or 200 fmol of standard peptides, 50 fmol of internal standard peptides and tryptic digest equivalent to 1 μg protein of E. coli lysate, and each injection for the whole cell lysate and crude membrane fraction samples included 50 fmol of internal standard peptides and tryptic digest equivalent to 1 μg protein of whole cell lysate and crude membrane fraction samples. The reason why we used E. coli digests for the calibration curve is to minimize the adsorption of the standard and internal standard peptides on the walls of tubes and pipette-tips; it is important to avoid loss of peptides while preparing the dilution series because of the quite low concentrations.

3. Multiplexed selected/multiple reaction monitoring (SRM/MRM) in nanoLC-TripleTOF5600 system.
The LC-MS/MS system consisted of a NanoLC-Ultra 2Dplus system (Eksigent Technologies, Dublin, CA, USA) coupled with a cHiPLC-nanoflex system (Eksigent Technologies) and a TripleTOF5600 (AB SCIEX, Framingham, MA, USA) equipped with a NanoSpray III ion source (AB SCIEX). Mobile phases A and B consisted of 0.1% formic acid in water and 0.1% formic acid in acetonitrile, respectively. The cleaned-up peptide samples were directly loaded onto a Nano cHiPLC 75 μm x 15 cm ReproSil-Pur C18-AQ 3 μm 120Å column (Eksigent Technologies) with injection amounts corresponding to 1 μg protein as described above, without a trap column. The peptides were separated and eluted from the column at a flow rate of 300 nL/min with a linear gradient as follows (mobile phase A:B): 100:0 for 40 min after injection for sample loading, 60:40 at 40 min, 10:90 at 41 min and up to 50 min, 98:2 at 50.1 min and up to 80 min. The eluted peptides were sprayed from a SilicaTip emitter (New Objective, Woburn, MA, USA) and positively ionized with the NanoSpray III ion source of the TripleTOF5600. The source/gas parameters were as follows: ion source gas 1 (20 psi), curtain gas (20 psi), ion spray voltage, floating (2300 V), interface heater temperature (150°C). Measurements of unlabeled and stable-isotope labeled peptides were acquired in the MRM-High Resolution (MRM-HR) mode, known as Parallel Reaction Monitoring (PRM) in the TripleTOF5600. The declustering potential was 80 V. The collision energy was optimized for each peptide. The collision energy spread was 0 V. The product ions were scanned with a MS/MS range of 100 to 1600 m/z. The accumulation time was 0.05 second for each target peptide. Auto calibration with β-galactosidase was performed every 4 runs.

MultiQuant software (AB SCIEX) was used for data analysis. The SRM/MRM transitions for each peptide consisted of the m/z value of the precursor ion and the m/z range of m/z ± 0.025 for several product ions. A peak was defined as positive when the signal-to-noise (S/N) ratio of peak intensity and the peak area were higher than 3 and 1000 counts, respectively, for the signal peaks detected at the same retention times as those of the internal standard peptides. When positive peaks were observed in one to five SRM/MRM transitions, we considered that the protein was expressed in the samples, and its protein expression level was calculated as described below.

4. Calculation of protein expression levels.
For each measurement, the target peptide was considered to exist in the sample if positive peaks were observed in one to five SRM/MRM transitions, and then the amount (fmol) of the target peptide was calculated from the peak area ratio (target peptide / the corresponding internal standard peptide) and calibration curve for each transition showing a positive peak. Subsequently, the protein expression level (fmol/μg protein) of the corresponding target protein in the whole cell lysate and crude membrane fraction samples was calculated for each transition by dividing the amount (fmol) of the target peptide by the total protein amount of whole cell lysate and crude membrane fraction samples examined (1 μg protein). Therefore, in one measurement, one to five values of protein expression level were obtained for each protein, and the average was calculated.

5. Calculation of the limit of quantification.
If no positive peak was observed, the protein expression level was defined as under the limit of quantification (ULQ). The LQ calculation was as follows: The peak area (counts) which gives the S/N ratio of 3 for peak intensity in the sample ($PA_{S/N3,sample}$) was calculated by means of eqn 1 from the intensity (cps) of background noise (I_{Noise}), and the peak area (counts) and intensity (cps) of internal standard peptide in the sample ($PA_{IS,sample}$ and $PI_{IS,sample}$, respectively). When $PA_{S/N3,sample}$ was more than 1000 counts, $PA_{S/N3}$,sample was used as the peak area for the calculation of LQ (PA_{LQ}). In contrast, when $PA_{S/N3,sample}$ was less than 1000 counts, 1000 counts was used as PA_{LQ}. When the calibration curve was obtained with eqn 2, the amount (fmol) of target protein equivalent to LQ ($A_{Target\ eq\ LQ}$) was calculated by means of eqn 3 from PA_{LQ}, $PA_{IS,sample}$ and the values of slope and intercept in eqn 2. Then, LQ was obtained with eqn 4 by dividing $A_{Target\ eq\ LQ}$ by the total protein amount of sample examined (A_{sample}, 1 μg protein).

$$PA_{S/N3,sample} = I_{Noise} \times 3 \times PA_{IS,sample} / PI_{IS,sample} \quad (1)$$

$$PA_{ST,Authentic} / PA_{IS,Authentic} = (Slope \times A_{ST,Authentic} + Intercept) \quad (2)$$

$$A_{Target\ eq\ LQ} = (PA_{LQ} / PA_{IS,sample} - Intercept) / Slope \quad (3)$$

$$LQ = A_{Target\ eq\ LQ} / A_{sample} \quad (4)$$

where $PA_{ST,Authentic}$ and $PA_{IS,Authentic}$ are the peak areas (counts) of standard peptide and internal standard peptide in authentic samples, respectively, and $A_{ST,Authentic}$ is the amount (fmol) of standard peptide in authentic samples.
